# Preventing Multiple Sclerosis: The Pediatric Perspective

**DOI:** 10.3389/fneur.2022.802380

**Published:** 2022-02-25

**Authors:** Duriel Hardy, Tanuja Chitnis, Emmanuelle Waubant, Brenda Banwell

**Affiliations:** ^1^Dell Children's Medical Center of Central Texas, Austin, TX, United States; ^2^Department of Neurology, Dell Medical School, University of Texas at Austin, Austin, TX, United States; ^3^Brigham and Women's Hospital and Harvard Medical School, Boston, MA, United States; ^4^Massachusetts General Hospital and Harvard Medical School, Boston, MA, United States; ^5^Weill Institute for Neurosciences, University of California, San Francisco, San Francisco, CA, United States; ^6^San Francisco Multiple Sclerosis Center, University of California, San Francisco, San Francisco, CA, United States; ^7^Center for Neuroinflammation and Neurotherapeutics, and Multiple Sclerosis Division, Department of Neurology, Perelman School of Medicine, University of Pennsylvania, Philadelphia, PA, United States; ^8^Division of Child Neurology, Department of Neurology, The Children's Hospital of Philadelphia, Perelman School of Medicine, University of Pennsylvania, Philadelphia, PA, United States

**Keywords:** multiple sclerosis, pediatric multiple sclerosis (MS), preventative medicine, demyelinating disease, MS environmental risk factors

## Abstract

Pediatric-onset multiple sclerosis (MS) is a predominantly relapsing-remitting neuroinflammatory disorder characterized by frequent relapses and high magnetic resonance imaging (MRI) lesion burden early in the disease course. Current treatment for pediatric MS relies on early initiation of disease-modifying therapies designed to prevent relapses and slow progression of disability. When considering the concept of MS prevention, one can conceptualize *primary prevention* (population- or at-risk population interventions that prevent the earliest facet of MS pathobiology and hence reduce disease incidence), or *secondary prevention* (prevention of disease consequence, such as reducing relapse frequency and lesion accrual, enhancing focal lesion repair, promoting CNS resilience against the more global facets of disease injury, and ultimately, preventing progression of neurological disability). Studying the pediatric MS population provides a unique opportunity to explore early-life exposures that contribute to the development of MS including perinatal and environmental risk determinants. Research is ongoing related to targeting these risk factors for potential MS *primary prevention*. Here we review these key risk factors, their proposed role in the pathogenesis of MS, and their potential implications for primary MS prevention.

## Introduction

Multiple sclerosis (MS) is a chronic demyelinating disease affecting the central nervous system that primarily affects adults; however, 3 to 10% of all patients diagnosed with MS experience their first demyelinating event prior to the age of 18 (1–3). In children, MS is characterized by a highly relapsing course though relatively complete or near-complete recovery from attacks is seen (4). Pediatric-onset MS patients face a lower risk for disability within the first 10 years of disease onset, and a longer time from onset to entry into secondary progressive disease compared to adult-onset patients. However, they develop disability overall at a younger age than when the disease starts in adulthood. Cognitive deficits, fatigue, and depression are also prominent features of pediatric-onset MS (1, 5) that impact quality of life. Current treatment strategies, particularly prompt initiation of highly effective disease modifying therapies, reduce relapses and may reduce the reduce relapses and reduce the likelihood of progressive disability.

The etiology of pediatric-onset MS is believed to be multifactorial involving a complex interplay between genetic and environmental risk factors (1, 6). The idea of studying early-life exposures and risk factors for MS to guide earlier targeted interventions against these risk determinants to prevent the disease is an important concept in the field. The most widely studied and most likely modifiable risk determinants associated with pediatric MS include environmental risk factors such as exposure to Epstein-Barr virus (EBV), low serum 25-(OH) hydroxyvitamin D /low sun exposure, diet and alterations in the gut microbiome, obesity, and exposure to cigarette smoking and other lung irritants (Table 1). Additionally, the role of perinatal risk factors in MS risk is still being evaluated. In this review we will discuss these key risk determinants and the potential therapeutic strategies to target these risk factors to prevent multiple sclerosis.

**Table 1:** Pediatric onset MS risk factors.

**Risk factor**	**Odds ratio or relative risk**	**Source(s)**	**Modifiable?**
Breast feeding	4.43 (OR) No association (12)	Cross-sectional, case control (7) Case control study (12)	Yes
EBV exposure	4.5 (OR)	Meta-analyses (14, 15)	Potentially
High serum 25-(OH) hydroxyvitamin D	0.72 (OR)	Meta-analysis/Mendelian Randomzation (16)	Yes
Sun exposure	Adjusted RR 0.55	Case control study (17)	Potentially
Gut-microbiome	Not available	Pilot cohort study (18), case control study (19)	Yes
Body Mass Index (BMI)	1.60 (OR, females) 1.42 (OR, males); 1.17 (OR)	Case control study (20) Meta-analysis (16)	Yes
Passive smoking	2.12 (RR)	Population-based case control study (21)	Yes
Air pollutants	Carbon monoxide OR 5.45 Sulfur Dioxide OR 3.99 Fine particulate matter OR 7.53	Multicenter case control study (22)	Yes
Household exposures	Rodenticides OR 2.10 Weed Control agents OR 1.99 Plant/Tree agents OR 2.72	Cross sectional analysis of case control study (23)	Yes

## Perinatal Risk Factors: The Role of Breast Milk

Breastfeeding is one of the earliest childhood exposures that has been investigated in relation to MS risk; however, its role in MS has yielded mixed results. In 2017, a small cross-sectional case control study performed at the University of Virginia analyzed the association of breastfeeding in infancy on future risk of pediatric onset MS by comparing 36 pediatric onset MS patients with 72 control patients (7). In this study, lack of breastfeeding was associated with a future diagnosis of pediatric onset MS (odds ratio-4.43; 95% confidence interval, *P* = 0.003) with 36% of the pediatric onset MS patients being breastfed compared to 71% of the controls. There have been other earlier studies to support the association between breastfeeding and decreased risk of MS (8, 9) as well as studies demonstrating a protective effect of breastfeeding against other autoimmune conditions such as Type 1 diabetes and inflammatory bowel disease (10). The hypothesized mechanism revolves around the idea that molecular mimicry between cow milk proteins and human self-antigens leads to the development of early MS. Thus, breast milk may provide protection from autoreactivity to cow milk (7). To support this hypothesis, there has been data suggesting increased abnormal T cell responses against certain cow milk proteins when compared to healthy controls in patients with type I diabetes, children with acquired demyelinating syndromes, and children with MS (11). Although this research provides compelling evidence for the benefits of breast milk, there was a large study by the US network of Pediatric MS centers that found no association of breast feeding with risk of pediatric MS (12). In this case control study, 265 pediatric MS cases were compared to 412 healthy controls, and after controlling for pregnancy-related factors, breast-feeding was not associated with MS (12). Nonetheless, for women that are able, breastfeeding may potentially reduce autoimmune dysregulation and has clear other benefits in the developing child including protection against infections, enhanced growth and development due to the balance of nutrients in breast milk, and improved brain growth and development (13). Early breastfeeding counseling to pregnant women, particularly to those who have a family history of MS, could be a potential preventative therapy against the development of MS while providing so many other benefits to both mother and child.

## Environmental Risk Factors: The Role of Epstein-Barr Virus (EBV) Exposure

There has been consistent evidence that past EBV infection is associated with increased risk of adult-onset multiple sclerosis including in meta-analyses (14, 15, 24–27). In a large national cohort study performed between 2004 and 2010 including 332 children, risk of MS was increased in children with past exposure to EBV (HR 2.04, 0.99–4.20) (27). Additionally, the US network of Pediatric MS centers published a large case control study that supported the association of remote EBV exposure and MS risk in children. This study found that EBV viral capsid antigen (EBV-VCA) seropositivity was associated with increased odds of having MS by 7.4 times (28). In children, it has been reported that up to 15% of children with an MS diagnosis are EBV-seronegative; however, a recent study demonstrated that some of these EBV-seronegative children carrying an MS diagnosis may not have been appropriately diagnosed. In this study, 25 EBV-seronegative patients among 189 pediatric patients diagnosed with CIS/MS were re-evaluated clinically, serologically and radiographically. Upon re-evaluation, 11 of 25 (44%) of these patients were found to be myelin oligodendrocyte glycoprotein (MOG) antibody positive, 4 of the remaining 14 patients did not meet 2017 McDonald criteria for MS, and of the 10 remaining patients who did meet 2017 McDonald criteria for MS had clinical features that were unusual for an MS diagnosis (29). Ultimately this study concluded that a diagnosis of pediatric MS is exceedingly rare in patients who are EBV-seronegative. There are numerous hypotheses that have been proposed to explain how EBV may be involved in MS pathogenesis: (i) EBV leads to chronic latent (and intermittently reactive) infection of human B cells, which then may prime T cells to cross-react and recognize CNS antigens via molecular mimicry; (ii) EBV may drive pro-inflammatory responses in latently infected B cells leading to expression of pro-inflammatory cytokines and reduction in anti-inflammatory cytokines (notably interleukin-10); and (iii) EBV may elicit “bystander damage” *via* induction of an antiviral immune response against infected cells in the CNS (30). With regards to EBV infection being a “preventable” risk factor, EBV vaccine research is underway, however the development of an EBV vaccine faces challenges including safety concerns due to oncogenic potential, lack of a suitable animal model for EBV disease, incomplete understanding of the exact route and mechanism of EBV infection, and concern that an EBV vaccine would not be commercially viable (30–32).

## Environmental Risk Factors: The Role of Sun and Serum 25-(OH) Hydroxyvitamin D Exposure

Decreased exposure to sunlight and low serum 25-(OH) hydroxyvitamin D levels have been implicated as important risk factors for the development of MS. Despite a large collection of data to support the association of both low sunlight and low serum 25-(OH) hydroxyvitamin D levels with MS risk, it has proven challenging to distinguish the independent effect from ultraviolet radiation (UVR) from that of 25-(OH) hydroxyvitamin D and vice versa as UVR is involved in the conversion of vitamin D into an active metabolite. There has been some work performed, however, that suggests independent effects on MS risk. In one early study performed in 2012, Baarnhielm et al. demonstrated that lower UVR exposure was associated with increase MS risk after correcting for serum 25-(OH) hydroxyvitamin D levels. In this population-based case control study, an increased MS risk was identified in those patients whom reported low UVR exposure (OR 2.2, 95% CI 1.5–3.3) and this association held true even after adjusting for 25-(OH) hydroxyvitamin D status (33). In a more recent study, sun exposure was examined over the life course of patients with and without MS. This study found that living in high ultraviolet-B (UV-B) areas before MS onset was associated with a 45% lower MS risk (adjusted RR 0.55, 95% CI 0.42–0.73) (17). Sunlight has been postulated to reduce MS risk through both 25-(OH) hydroxyvitamin D independent and dependent pathways. Independent from vitamin D, it has been proposed that sun exposure may lead to suppression of cell mediated immunity as well as modulating the release of cytokines and chemokines (34). The effects of UVR exposure has been studied in experimental autoimmune encephalomyelitis (EAE) models, our current model of MS in mice, showing a reduction in peripheral inflammation in these mice after UVR exposure (35). “Prescribing” sun exposure as a preventative measure for MS is challenging as the amount of sunlight, duration, and timing of exposure are unknown. Additionally, risk of skin cancer from this exposure presents safety concerns.

Serum 25-(OH) hydroxyvitamin D levels have been an extensively studied risk determinant for the development of MS. Several studies have discovered not only an association of serum 25-(OH) hydroxyvitamin D levels with risk of developing MS, but some studies have even proposed a causal role for low 25-(OH) hydroxyvitamin D in the pathogenesis of MS (16, 36, 37). In one such study, Mendelian randomization, an analysis that uses genetic associations to test the effects of biomarkers on the risk of a disease, was used to identify a potential causal relationship between serum 25-(OH) hydroxyvitamin D levels and risk of pediatric-onset MS. In this study, meta-analysis showed increasing levels of serum 25-(OH) hydroxyvitamin D (based on a vitamin D genetic risk score constructed using 3 single nucleotide polymorphisms associated with vitamin D levels) decreased the odds of pediatric-onset MS (for each additional risk SNP OR = 0.72, 95% CI: 0.55–0.94; *P* = 0.02) after controlling for sex, genetic ancestry, HLA-DRB1^*^15, and more than 100 single nucleotide polymorphisms identified as MS-risk variants (16). In a separate large prospective Canadian cohort study, it was also shown that baseline serum 25-(OH) hydroxyvitamin D status at the time of an incident demyelinating attack was associated with likelihood of further relapses confirming a diagnosis of pediatric-onset MS (27). More specifically, this study showed that a 10 mmol/L decrease in 25-(OH) hydroxyvitamin D was associated with a 20% relative increase in risk of pediatric MS compared to monophasic demyelination (*p* = 0.006) (27). Similar studies performed in adult patients with MS support a strong association of serum 25-(OH) hydroxyvitamin D and MS risk (36–38). One particular adult study, using data from the Finnish Maternity Cohort, showed that a 50 nmol/L increase in 25-(OH) hydroxyvitamin D was associated with a 39% reduced risk of MS (RR 0.61, 95% CI 0.44–0.85), *p* = 0.003. Additionally, it was shown that MS risk was 2-fold higher in women with 25-(OH) hydroxyvitamin D <30 nmol/L as compared to women with 25(OH)D ≥50 nmol/L (RR 2.02, 95% CI 1.18–3.45, *p* = 0.01) (38). The precise time in which 25-(OH) hydroxyvitamin D may exert its greatest influence on the risk of MS is unknown; however, there is evidence that lower serum 25-(OH) hydroxyvitamin D levels as early as the neonatal period are associated with a higher risk of MS. The influence of 25-(OH) hydroxyvitamin D in neonates was evaluated in two studies. In a Danish case-control study utilizing the Danish Newborn Screening Biobank (DNSB) and the Danish MS registry, MS risk was highest among patients with the lowest neonatal serum 25-(OH) hydroxyvitamin D levels (39). In a cohort study including 199 cases of MS, a 38% lower risk of MS was observed in women whose mothers drank 2–3 glasses of milk during pregnancy compared to mothers that drank little to no milk. This study also observed that daughters of mothers with higher 25-(OH) hydroxyvitamin D intake or predicted serum 25-(OH) hydroxyvitamin D levels during pregnancy had a lower risk of developing MS suggesting that 25-(OH) hydroxyvitamin D insufficiency can exert its effect as early as the in utero-stage of life (40). Similarly, in a prospective, nested case control study utilizing the Finnish Maternity Cohort (FMC), it was demonstrated that levels of 25(OH) hydroxyvitamin D that were greater than or equal to 75 (vs <75) nmol/L in women during pregnancy (collected between 10 and 14 weeks) were associated with a decreased risk of MS [odds ratio (OR) 0.39, 95% confidence interval (CI) 0.16–0.98] (41). There have been two studies that did not show an association between pregnancy/early neonatal vitamin D levels and future MS risk (42, 43). The first study, utilizing the Northern Sweden Maternity Cohort, showed there was no association between maternal 25-(OH) hydroxyvitamin D levels and risk of MS in offspring (42), however this study had a very small sample size and the association identified in this study had a large confidence interval making the results challenging to interpret. The second study, was a Swedish population case control study that evaluated neonatal blood samples used for phenylketonuria (PKU) to compare MS cases and controls. In this study it was shown that there was no association between neonatal 25-hydroxyvitamin D quintile and risk of multiple sclerosis (crude odds ratio = 1.0, 95% confidence interval = 0.68–1.44, for the highest quintile compared to the lowest) (43), however this study is limited in that some of the older samples showed evidence of 25-(OH) hydroxyvitamin D degradation which may have contributed to the null findings. There was also very low overall control participation in this particular study.

From a mechanistic standpoint, 25-(OH) hydroxyvitamin D is believed to attenuate the T-cell response to autoantigens, suppress the production of pro-inflammatory cytokines such as interferon-gamma and TH-17-interleukin 17, and increase T-regulatory cells (39, 44). Thus, this evidence suggests an important role of 25-(OH) hydroxyvitamin D in the development of MS, and one might propose implementation of 25-(OH) hydroxyvitamin D supplementation to individuals at higher risk of developing MS, such as those with a family history. Moreover, given evidence implicating a role for 25-(OH) hydroxyvitamin D as early as in utero, we might propose implementing 25-(OH) hydroxyvitamin D in at risk patients during pregnancy and/or at birth/infancy. 25-(OH) hydroxyvitamin D is a relatively safe and cost-effective therapy (45, 46) that has the potential to prevent MS, and like breast milk, has other potential health benefits.

## Environmental Risk Factors: The Role of Diet and The Gut-Microbiome

Diet plays an important role in the prevention of cardiovascular disease, stroke, and diabetes (47, 48), and emerging data suggests that diet and modulation of the gut microbiome may also influence the risk of pediatric MS. Specific dietary factors, other than vitamin D, have been investigated to identify potential roles in risk of MS. These studies may be confounded however due to reliance on patient/parents' recall of diet specifics and non-specific questionnaires. One case control study comparing 312 POMS cases with 456 controls, found that iron consumption below the recommended guidelines was associated with an increased risk of MS (odds ratio = 1.80, *p* < 0.01). This study also evaluated for associations between other dietary factors such as intake of fats, proteins, carbohydrates, sugars, fruits or vegetables; however, no significant difference in intake was identified between cases and controls (49). One case-control study (170 cases, 331 controls) has explored associations between sodium intake and pediatric MS. This study did not find an association between higher sodium intake and risk of POMS (50).

The role of the gut microbiome in CNS autoimmunity has become increasingly recognized (51). Studies have revealed that patients with MS may have a different gut microbiome composition compared to healthy controls suggesting modulation of the microbiome may help prevent MS (18, 52). In animal models, modulation of the gut microbiome appears to influence risk and severity of EAE (53–55) including two studies that showed germ-free mice (mice free of microbes) were less likely to develop EAE (53, 54). In a case-control study comparing children with new-onset MS and healthy controls, a significant increase in relative abundance for members of the *Desulfovibrionaceae* (*Bilophila, Desulfovibrio* and *Christensenellaceae*) and depletion in *Lachnospiraceae* and *Ruminococcaceae* in stool samples (all p and q < 0.000005) was identified in the children with MS irrespective of exposure to disease-modifying therapy. The changes identified in the microbiota of the pediatric MS patients support the idea that an increase in pro-inflammatory microbiota and a decrease in anti-inflammatory microbiota contribute to the early immune dysregulation seen in MS (19). Furthermore, differences in the relationships between immune markers and gut microbiota have been observed when comparing children with MS and control cases (18). In an additional case control study utilizing the same cohort of patients described above, children without MS were found to have an inverse correlation between gut microbiota evenness and Th17 and Th2 blood markers, with microbiota dominated by specific taxa being associated with decreased immune markers. This effect was lost in children with MS. Additionally, at the phylum level, Bacteroides was inversely associated with Th17 in children with MS (r = 0.719, *p* = 0.008) but not controls (r = 0.320, *p* = 0.401). Alternatively, Fusobacteria was found to be positively correlated with T-regulatory cells in controls (r = 0.829, *p* = 0.006) but not in children with MS (r = −0.069, *p* = 0.808) (18). With regard to the potential function of gut microbiota in the pathogenesis of MS, evidence suggests, utilizing a validated algorithm (56), that enrichment of microbial genes involved in glutathione metabolism was observed in MS cases rather than control cases (19). Disruption of glutathione homeostasis has previously been reported as a possible mechanism of MS neurodegeneration (57).

Taken together, focusing on dietary modifications, adequate iron intake and diets that influence the microbiome in an “anti-inflammatory” manner may be plausible approaches to reduce the risk of MS in in children with a family history of MS. Further studies will be required to identify specific diets that alter the microbiome in a favorable manner by potentially increasing anti-inflammatory microbiota and decreasing pro-inflammatory microbiota. Additionally, it will be important to identify whether microbiome changes occur prior to onset or as a result of MS or initiation of MS therapies or some combination of these factors. This will be important to understand to determine the most optimal timing for implementation of potential preventative gut microbiome intervention.

## Environmental Risk Factors: The Role of Obesity

Several studies have provided evidence that elevated BMI is associated with increased risk of childhood-onset MS (20, 58, 59). Elevated BMI has been associated with increased risk of pediatric MS in both post-pubertal girls (OR = 1.60, 95% confidence interval [CI]: 1.12, 2.27, *P* = 0.009) and boys (OR = 1.43, 95% CI: 1.08, 1.88, *P* = 0.011) in a case-control study of 254 pediatric-onset MS cases and 420 controls (20). An association of obesity and pediatric-onset MS was also suggested in a large meta-analysis of a US and Swedish cohort of POMS using BMI genetic risk scores (GRS) that incorporated the cumulative effect of 97 variants associated with BMI (16). Additionally, in a large longitudinal retrospective analysis of prospectively collected data, of 774 POMS cases, elevated BMI z-scores were associated with increased risk of MS in both girls ages 7-13 (HR 1.17 to 1.21) and boys ages 8–10 (HR 1.14 to 1.15) (58).

There have been several proposed mechanisms through which obesity may contribute to the pathogenesis of MS. The presence of increased adipose tissue hormone leptin is believed to play a role in the risk of MS given its pro-inflammatory properties (60). Animal models have also supported this mechanism demonstrating that leptin-deficient mice failed to develop EAE when stimulated with MOG 35-55 specific T-cells coinciding with decreased levels of pro-inflammatory cytokines (IL-2, IL-6, INF-γ, TNF-α) (61). Obesity has also been associated with IL-6 dependent TH17 production which has been shown to exacerbate EAE in mice (62). Another adipokine, adiponectin, has also been implicated in the pathogenesis of MS (63). One study found elevated levels of leptin and fatty acid binding protein-4 as well as reduced adiponectin in boys and girls with MS compared to age and sex-matched controls (64). A recent study comparing 33 children with MS to 54 children with MOGAD, and 29 healthy controls, observed significantly higher levels of adiponectin in the serum of MS patients compared to both the MOGAD and healthy control patients (*p* = 0.02) (59). This study also investigated the functional consequence of elevated adiponectin on immune cells and discovered that adiponectin from the serum of pediatric MS patients led to pro-inflammatory responses in CD14+ monocytes, T-cell activation, upregulation of CNS microglia pro-inflammatory markers, and downregulation of CNS microglial specific quiescent/anti-inflammatory markers (63) all evidence supporting a role of adiponectin to induce disease in children with MS. Finally, there has been evidence suggesting that obesity, through disruption of TH17/Treg balance, may alter the gut microbiome resulting in dysregulation of the intestinal immune response (65).

Promoting healthy weight in children is an intervention that should be enforced in all youth regardless of their potential MS risk. The benefits associated with healthy weight are plentiful, and with the added potential of possibly preventing a chronic neuroinflammatory disorder like MS, it is truly an essential therapeutic strategy. Additionally, the pathway to achieve healthy weight typically involves exercise and a healthy diet, two additional interventions that provide immense overall health benefits. Exercise alone, though beyond the scope of this review, has proven to have potential neurologic benefits including enhancing the production of neuroprotective trophic factors, promoting neuronal survival, promoting oligodendrocyte proliferation and repopulation, reducing neuronal injury, astrogliosis and modulation of cytokine production (66–68). In summary, counseling patients on the importance of a healthy lifestyle may have a significant impact on overall health and possibly contribute to the prevention of MS.

## Environmental Risk Factors: The Role of Exposure To Cigarette Smoking and Other Air Pollution

Exposure to cigarette smoking, both active and passive, is another risk determinant that has consistently been identified to be associated with the risk of MS (21, 69, 70). As early as the 1960's, an association between active smoking and development of MS has been reported (71). More recently, in 2009, active smoking was identified as a risk factor for MS in a large European multi-national case-control study where the odds of MS in smokers was 1.5 (95% CI 1.3–1.8) in Sweden; 1.8 (95% CI 1.1–2.9) in Norway; and 1.3 (95% CI 1.0–1.9) in the UK (72). In children, where active smoking is generally less common (or at least, less commonly reported), the association of exposure to passive cigarette smoking and MS risk has been explored instead. In a population-based, case-control study conducted in France, the association of passive cigarette smoking exposure and risk of MS was assessed in 129 POMS cases and 1,038 age-matched controls and found that passive exposure to parental smoking was associated with increased risk of MS (relative risk (RR) 2.12, 95% CI 1.43–3.15). An increased risk of MS was significantly associated with longer duration of passive smoke exposure (RR 2.49, CI 1.53–4.08) (21). In another study, an association between secondhand smoke exposure and MS was evaluated in a cohort of 216 children with monophasic demyelination and 81 children with MS and found that secondhand smoke exposure was not an independent risk factor for the development of MS, but when combined with the presence of HLA-DRB1^*^15, the odds of MS significantly increased [odds ratio (OR) = 3.7; 95% confidence interval (CI): 1.17–11.9] suggesting a gene-environment interaction (70).

The major proposed mechanisms of exposure to cigarette smoking and development of MS include direct neurotoxicity, demyelination and immune modulation (21, 65, 70, 71). Cyanide, a component of cigarette smoke, has been shown to cause demyelination in rat models (69). Cigarette smoke may also be involved in the pathogenesis of MS through modulation of cellular and humoral immune responses (73). Smoking promotes T-cell activation and proliferation in the lungs and thus may contribute to increased immune activation (74). Given this association of both active and passive smoking with the development of MS, one could postulate that simply counseling patients (and housemates and/or parents) to avoid cigarette smoking could prevent the development of MS in many young individuals. This benign intervention may also have a beneficial effect on the smoker's overall health and the health of those around them.

In addition to cigarette smoke, air pollution and lung irritants such as fine particulate matter, carbon monoxide, and sulfur dioxide have been evaluated for associations with MS risk. In a multicenter case-control study performed in 2018, it was shown that fine particulate matter, carbon monoxide, sulfur dioxide and lead air emissions were associated with increased odds for pediatric MS (*P* < 0.01) (22). Similar work evaluating household chemical exposures and MS risk also demonstrated evidence for exposure to rodenticides (OR 2.10), weed control agents (OR 1.99) and products for plant/tree disease control (OR 2.72) to be associated with increased MS risk in childhood (23). There has been some conflicting evidence however, with some studies including a study using the Nurses' Health Study showing that particulate matter was not statistically associated with MS risk (75). Nonetheless lung irritants are exposures that can be potentially prevented ad thus warrants further investigation. Proposed mechanisms for how these irritants can influence MS risk include release of pro-inflammatory cytokines, promotion of oxidative stress, and stimulation of the immune response to activate auto-aggressive T cells to enter the central nervous system (22, 76).

## Discussion

Although the precise etiology of pediatric-onset MS has yet to be identified, it has become increasingly more evident that multiple risk determinants may play a crucial role. Investigating these environmental determinants in the pediatric-onset MS population allows one to evaluate the earliest influences on MS development and pathogenesis in individuals that are temporally closer to the biologic inciting event(s) of the disease. Moreover, studying risk determinants in this population eliminates some of the challenges with recall bias, as children are temporally closer to the incident exposures being studied.

Some of the risk determinants associated with POMS may be modifiable at the population level to potentially prevent disease onset. Implementation of sun exposure and 25-(OH) hydroxyvitamin D supplementation as well as recommendations for healthy diet and avoidance of exposure to cigarette smoke are simple, yet potentially effective strategies to not only improve general health, but to also reduce risk of MS. The possibility of an EBV vaccine is intriguing; however, much research will be required to create a vaccine that is safe and effective. The question of whom and when to enforce these strategies remains unclear. We propose based on the current literature that patients with a genetic predisposition to MS (i.e., first-degree relative with MS), may be the population that would benefit the most from these recommendations. In regard to timing of these interventions, we propose initiating these recommendations when planning conception and throughout gestation, childhood and adolescence. Ultimately these interventions are safe with low potential for adverse effects, and notably, could also have other health benefits including, but not limited to, improved cardiovascular health, pulmonary health, mental health, and energy. Further studies, specifically in the form of randomized clinical trials, will be required to provide more definitive evidence of exactly whom, when, and how much sun exposure/ 25-(OH) hydroxyvitamin D supplementation should be given for primary prevention of MS. Another question is how might we measure the effect of implementing these strategies in neonates? Answering this question will require collaborative efforts to compare incidence of pediatric MS diagnoses in those individuals provided with these interventions vs. absence of these interventions.

## Author Contributions

DH, TC, EW, and BB all contributed to the conception, organization, and content material that was included in this manuscript. DH wrote the first draft of the manuscript. All authors contributed to manuscript revision and have read and approved the final version of this manuscript.

## Conflict of Interest

The authors declare that the research was conducted in the absence of any commercial or financial relationships that could be construed as a potential conflict of interest.

## Publisher's Note

All claims expressed in this article are solely those of the authors and do not necessarily represent those of their affiliated organizations, or those of the publisher, the editors and the reviewers. Any product that may be evaluated in this article, or claim that may be made by its manufacturer, is not guaranteed or endorsed by the publisher.
